# Classification and counting of cells in brightfield microscopy images: an application of convolutional neural networks

**DOI:** 10.1038/s41598-024-59625-z

**Published:** 2024-04-19

**Authors:** E. K. G. D. Ferreira, G. F. Silveira

**Affiliations:** Carlos Chagas Institute, Curitiba, PR CEP 81310-020 Brazil

**Keywords:** Cell lineages, CNNs, Machine learning, Microscopic image, Cell biology, Biotechnology

## Abstract

Microscopy is integral to medical research, facilitating the exploration of various biological questions, notably cell quantification. However, this process's time-consuming and error-prone nature, attributed to human intervention or automated methods usually applied to fluorescent images, presents challenges. In response, machine learning algorithms have been integrated into microscopy, automating tasks and constructing predictive models from vast datasets. These models adeptly learn representations for object detection, image segmentation, and target classification. An advantageous strategy involves utilizing unstained images, preserving cell integrity and enabling morphology-based classification—something hindered when fluorescent markers are used. The aim is to introduce a model proficient in classifying distinct cell lineages in digital contrast microscopy images. Additionally, the goal is to create a predictive model identifying lineage and determining optimal quantification of cell numbers. Employing a CNN machine learning algorithm, a classification model predicting cellular lineage achieved a remarkable accuracy of 93%, with ROC curve results nearing 1.0, showcasing robust performance. However, some lineages, namely SH-SY5Y (78%), HUH7_mayv (85%), and A549 (88%), exhibited slightly lower accuracies. These outcomes not only underscore the model's quality but also emphasize CNNs' potential in addressing the inherent complexities of microscopic images.

## Introduction

Microscopy was invented in the early seventeenth century. Since then, the method has been perfected and now plays a fundamental role in healthcare^1^. Microscopy images are a rich source of biological data^2^, which allow users to observe the simplest to the most complex structure, increasing our understanding of the vast level of cell heterogeneity in areas including cellular composition, structure, and morphology, all of which are related to cell physiology^3^, proliferation, and cell death among other factors. It is common practice in the laboratory to count cells present in cell culture. However, quantification can be a time-consuming and error-prone process since most microscopy procedures require a human operator or the use of automated image processing methods, which are usually applied to fluorescence-labeled images.

Currently, there has been a growth in the number of applications for learning models in many areas of biological research^4^. Machine learning algorithms, using automated processing, have been applied to microscopy in order to circumvent the problems described above^5^. These models are surprisingly capable in terms of cell segmentation/tracking, morphological analysis, and noise reduction^6,7^.

One of the potential uses of this tool is to generate predictive models using a large amount of data, allowing it to learn representations, which can be applied to object detection, image segmentation, or target classification^2–8^.

CNN (Convolutional Neural Network), a deep learning algorithm, stands out above all others and, in deep learning, is part of the class of machine learning techniques that use multilayer artificial neural networks for automated data analysis^9,10^ and it is often used for image data^2^. It functions similarly to a brain by emitting a signal and then receiving a stimulus in response. The CNN algorithm uses kernels (filters) in the convolutional layers; they begin working randomly and can be trained to learn how to perform specific tasks using supervised (when the target is known) or unsupervised machine learning techniques^11^. Compared to other machine learning algorithms, CNN has the advantage of extracting features from the image, simplifying and avoiding image preprocessing, and improving the validity and accuracy of detection^12,13^. It can be used for classification purposes, whereby the model learns the characteristics of the images and classifies them according to their label. For our study, our labels are eight different cell lines.

This study is a continuation of a previous investigation into the quantification of cells in microscopy images^14^. Cell image quantification is fundamental in many biological and medical research tasks. However, to gain a more complete and detailed understanding of the cellular environment, it is necessary not only to quantify the cells but also to classify them according to their specific lineages. In this article, we expand the scope of the previous study by focusing on creating a model capable of classifying images according to each identified cell line. The objective was to propose a model that is able to identify different cell lines in digital contrast microscopy images using their morphology and present us with the best predictive model that quantifies the number of cells that are present in these images. In addition to not damaging the cell culture with chromogens, the advantage of using unstained images allows the model to learn to classify the images using their morphology, which would not be possible if, for example, the images contained a nuclear marker. The main application is to assist in scientific research experiments. In everyday life, researchers need to identify the lineage present in generated images by analyzing different lineages in multiple images. This identification is essential to determine the most appropriate algorithm to be used later in the counting of the cells present in the image. The accuracy and performance of the developed classification algorithm offer new possibilities for studies that require the precise identification of cell lines in microscopy images, opening doors for further advances in biological and biomedical research.

Most of the platforms that carry out the cell quantification process work with the pre-processing of images, with the need for labeling of cellular components by fluorescence. Additionally, some of the solutions that have different approaches using brightfield, require the images to be acquired and analyzed by the platform itself, which makes the technology less accessible. In this study, we sought to demonstrate the ability of a relatively simple CNN model to qualify different cell line images in digital contrast microscopy, as a step prior to quantifying the number of cells present in these images. This approach has advantages over direct quantification, as not all strains have sufficient characteristics for the regression process. In addition, it is not necessary for classification or regression to pre-label the cells, which reduces the presence of artifacts in the images. The solution presented in the manuscript has the potential to be used in any quality image, acquired under any microscope.

### Related work

The long history of pathology encompasses the morphological classification of cells and tissues at a subcellular level, observed through the microscope. Subcellular features, such as increased nuclear-cytoplasmic ratio, granular cytoplasm, and a prominent round nucleus with a distinct nucleolus, are influenced by subcellular organelles. This spans fields such as cytology and histology^15^.

When diagnosing and classifying diseases, doctors identify patterns in microscopic images, interpreting their significance based on past experience. In cell biology, cytology, and pathology, enhancing the identification and analysis of cellular or tissue characteristics can occur in two ways: through staining with dyes or marking molecules with fluorescent light; or through optical filtering in dark-field or bright-field microscopy, including label-free images such as phase contrast and differential interference contrast. The first method describes subcellular features, such as the distribution of specific proteins or molecules. The second method describes characteristics as a map of the refractive index of various proteins or molecules^16^.

Since the early 2000s, several machine learning-based computational strategies have been proposed to distinguish between cell types and states through microscopy^17–19^. Machine learning automates and optimizes cell classification based on quantitative metrics. The combination of pattern recognition and machine learning is opening new frontiers not only in industry but also in biomedical and medical Imaging^16^. The use of such algorithms could assist pathologists and scientists by reducing the time spent on manual image assessment, minimizing human error, and making the evaluation of large datasets of images feasible. A general approach using quantitative image parameters as predictors involves tabulating predictors with known classifiers in a training dataset, standardizing parameters, reducing data through principal components or a similar technique, assessing algorithm performance through cross-validation on the training dataset, and then applying the trained algorithm to a naive dataset to determine predictive power^20^.

## Results

### The model was correct in most of the lineages

**Figure 1:**
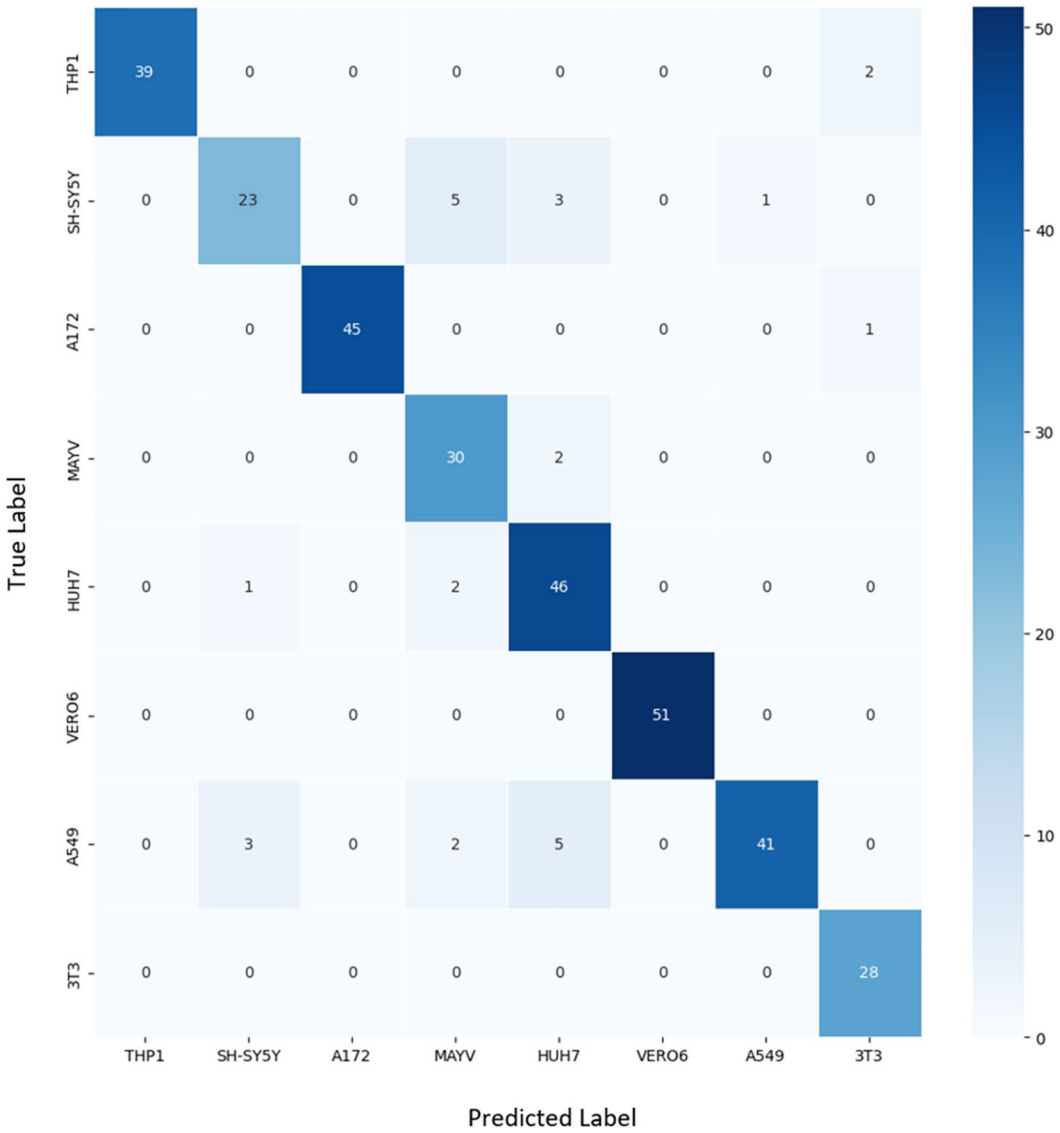
Confusion matrix plotted on color map. In the diagonal line is presenting the TP of each lineage and the other values present FP.

After launching the proposed algorithm with the model that had already been trained with the validation images (10% of the images), the confusion matrix (Fig. 1) presented most of the images corresponding to its class (TP). The model correctly identified all the images of the VERO6 and 3T3 lineage. For the other strains, it made errors in fewer than six images (FP).

### Accuracies of over 86% were obtained for five strains

**Figure 2:**
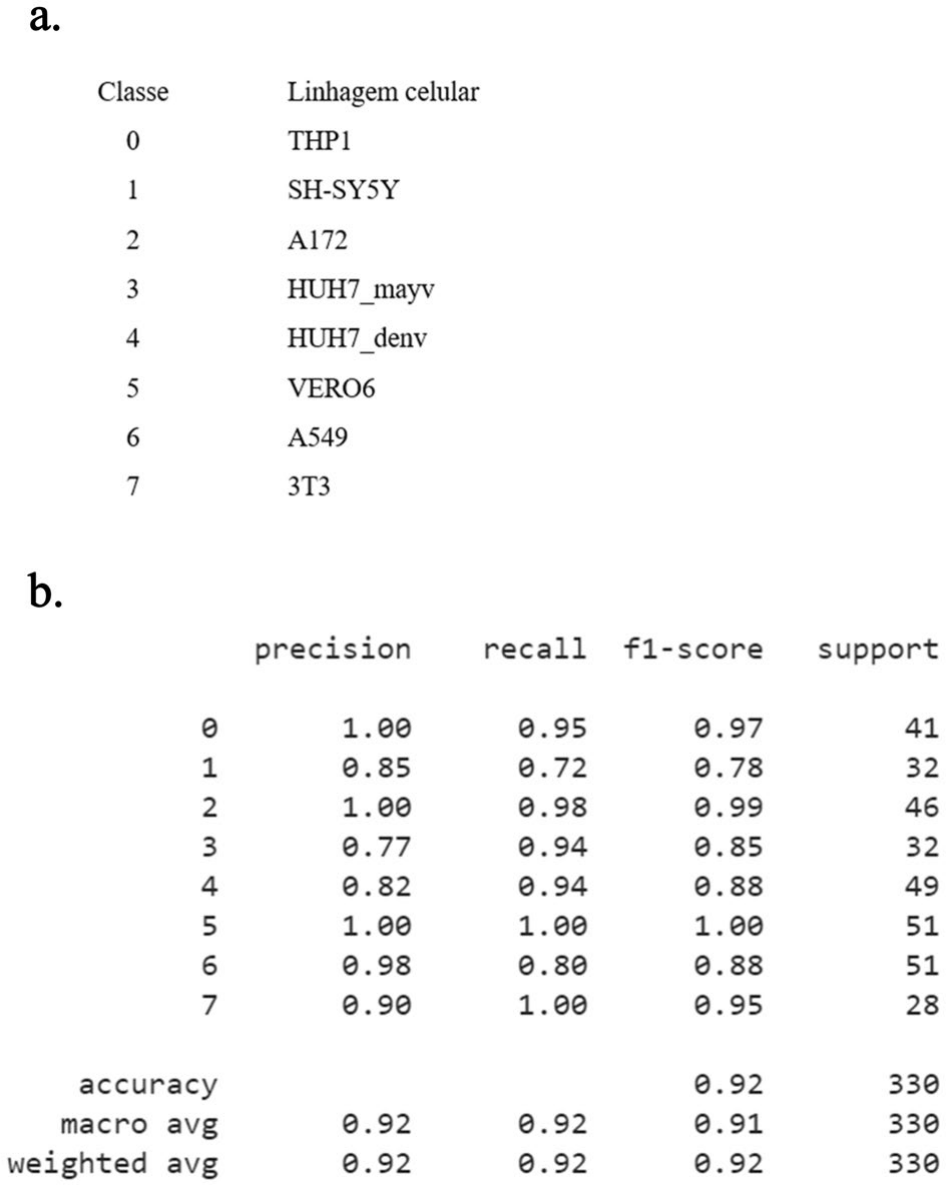
Metrics for the evaluation of the accuracy of a classificatory model. (**a**) In Precision measures the proportion of TP. In Recall evaluates the ratio of correct positive examples to the total of actual positives. (**b**) In F1 Score, it combines Accuracy and Recall into a single score, providing an overall measure of model performance.

The precision, recall, and F1-score were calculated for each strain, and we saw that the least accurate results were obtained for the SH-SY5Y, HUH7_mayv, and HUH7_denv strains (Fig. 2a). Even after applying filters, their accuracy was lower than 86% for the parameters above. In comparison, an F1-score of 97% was achieved for the THP strain, 99% for the A172 strain, and 100% for the VERO6 strain (in general, this cell line showed more accurate results). Similarly, the accuracy of the 3T3 lineage was above 95% (Fig. 2b).

### At least five strains presented ROC curve = 1.0

**Figure 3:**
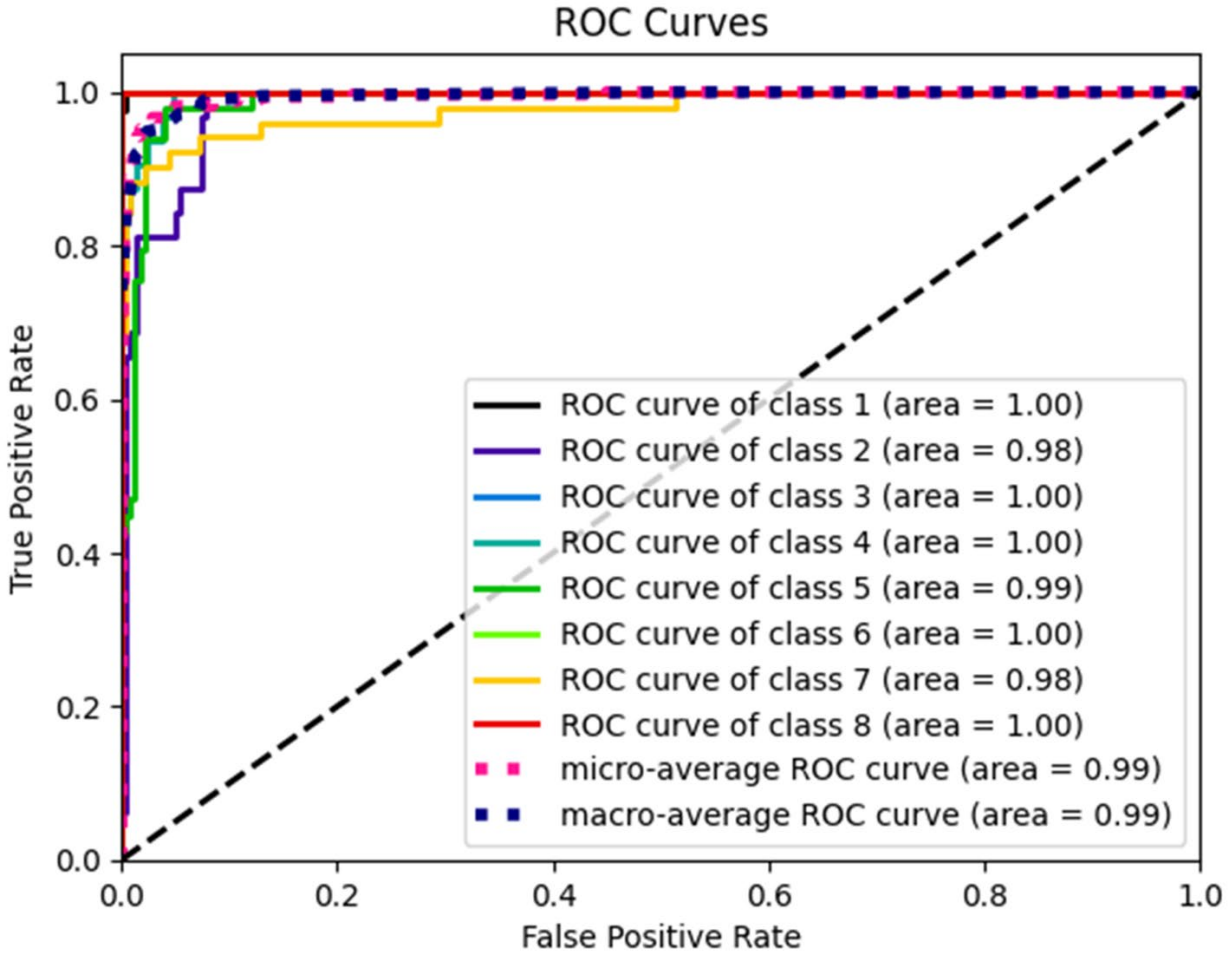
Plotting of the ROC curve for the eight strains analyzed. Each curve in the graph represents one of the lineages classified by the model.

The ROC curve was plotted to assess the model's sensitivity and classify it correctly. From the curves, it is possible to observe that in all lineages, the values were close to or equal to 1.0, suggesting that the model is able to classify each of the analyzed lineages (Fig. 3).

### Among the eight strains analyzed, A172 presented the lowest error in the regression model

In order to analyze the accuracy of the regression models in different scenarios, the models of each lineage were compared for comparison (Table 1). The strains with the highest error were 3T3, with MSE equal to 29,761.49, VERO6, with 13,055.47, and HUH7_denv, with 12,672.81. The strains with the lowest error were A172, with MSE of 493.93, SH-SY5Y, with 3,635.91, HUH7_mayv, with 4,628.09, THP1, with 5,302.80, and A549, with 5,406.52.

**Table 1:** Performance comparison of regression models among eight strains analyzed.

Lineages	Total images	Training (70%)	Test (30%)	R2 score	MAE	MSE
THP1	1.527	1.068	459	84.39%	50,52	5.302,80
SH-SY5Y	1.052	736	316	91.26%	45,71	3.635,91
HUH7_mayv	1.152	806	346	87.16%	53,38	4.628,09
A172	1.515	1.060	455	87.6%	16,79	493,93
HUH7_denv	1.368	957	411	90.29%	81,28	12.672,81
VERO6	1.613	1.129	484	92.7%	83,18	13.055,47
A549	1.023	716	307	93.01%	56,21	5.406,52
3T3	748	523	225	− 2.77%	129,9	29.761,49

## Discussion

Counting bright field microscopy images (digital contrast) is a challenging task due to the low level of differentiation in images between the background and cells^21^^.^ In addition, quantification is an important step in biological analyses, Oswal et al.^22^ pointed out that, previously, pathologists used to perform most of these activities, such as manually counting the total and abnormal cells. However, these manual methods were time-consuming and tended to generate inconsistent results due to human error. With the automation proposed in our previous research, it was possible to quantify the number of cells present in digital phase contrast images, and it was also possible to correctly classify these images in order to produce better results.

This study does not include binary classification with unbalanced classes, so the F1-score became a more significant metric. In the worst case, the proposed strategy gave an F1-score of 78%. When this metric is low, it means that either the recall, precision, or neither have produced satisfactory results. In our case, a result of 78% is not too low. In similar work^21^, Uka et al. showed an average accuracy of 78% for similar imaging in the study counting cells in low-contrast microscopic images. We obtained an average accuracy of approximately 90%. The least accurate classification result was obtained for the SH-SY5Y strain (78% F1-score); however, the quantification model presented satisfactory results, with low MSE (3,635.91). The most important metric for this result was the recall. In this case, the proportion of true positives in relation to the total positives was evaluated. This could easily be inferred by looking at the proportional errors in the confusion matrix. However, the strategy presented an F1-score of around 90% or higher for all other strains. In the case of the VERO6 lineage, the method could classify 100% of the images correctly. This highlights the possibility of correctly classifying the most challenging clear field cell lines by computational means. By working non-destructively through artificial intelligence, in addition to reducing evaluation costs (without the use of contrast markers), this strategy allows for reproducible and reliable automatic evaluation. Analyzing the strains in isolation, the model had worse classification performance in SH-SY5Y (neuroblastoma)^23^, HUH7_mayv (liver cell treated with Mayaro virus)^24^, HUH7_denv (liver cell treated with dengue virus)^24^ and A549 (lung epithelial cell)^25^. This may have occurred due to the morphological similarity of these cells, in the case of lineages of the epithelial type of coating (they constitute the coating that surrounds all the internal and external surfaces of the organs)^26^, which was observed in the images, even having origin in different tissues. The HUH7 strain produced similar F1-scores. Despite a low classification accuracy, the HUH7 strain treated with the Mayaro virus gave a low error in the regression model, with an MSE = 4628.09. Similar results were obtained with SH-SY5Y.

Using preprocessing steps to maximize the images' specific and interesting characteristics proved productive and advantageous. The filters may vary according to the classification's purpose, but the gain brought by this processing was clear. When visualizing these lineages on the ROC curve, which is then constructed by plotting the TPR (sensitivity) as a function of the FPR (specificity) at different classification threshold values, the area under the curve (AUC) is often used as a single measure of model performance. According to Perez (2021), the higher the AUC, the better the model's performance. AUC values close to 1 indicate a good performance of the model, while values close to 0.5 indicate a performance similar to that of a random classifier^27^^.^

## Materials and methods

The present work was an offshoot of a previous work of the group published in Scientific Reports, in which we used CNN to quantify the number of cells present in the microscopy images^14^. Our regression algorithm showed good performance and accuracy in two of the three strains tested, demonstrating that not all cells can be equally quantified by this technique. Thus, we present in the present manuscript the development of a model capable of identifying which cell lineage is present in each image based on a classification algorithm. CNNs are widely used for image data, being configured through convolutional layers, which apply filters to detect specific features in image regions. These traits are then combined and processed into subsequent layers, including pooling and fully connected layers, to perform tasks such as classification, object detection, or segmentation. Despite being a "simple" construction model, it was able to solve the problem and, therefore, no complex modifications were necessary.

### Image database

The used images were acquired in projects analyzed by the Harmony software (version 3.5), embedded in an automated microscopy High Content Screening (HCS). Only phase contrast images were selected. The images of the A549, HUH7_denv, 3T3, VERO6, THP1, SH-SY5Y, A172 and HUH7_mayv cell lines were used. Light contrast adjustments (highlighting the nuclear marking) and background correction (setting the image's background) were performed in Harmony.

### Processing environment

We used Google Colab’s Integrated Development Environment (IDE) due to its large memory (currently available with 12.72 GB RAM and 107.77 GB HD). For processing purposes, we imported several libraries from the Python v9 programming language. Data (including all data, unique materials, documentation, and code used in analysis) is available at Dataset: Ferreira, E. K. G. D. & Silveira, Guilherme F. 2023. “Data-Analysis-Laboratory/Microscopy-Image-Analysis-Classification-Script-Article: 1.0.0”. Zenodo. 10.5281/zenodo.8415315, accessible at the link: https://zenodo.org/badge/latestdoi/701446984.

### Segmentation and increase of the image bank

The Data Augmentation technique was used to increase the number of images in the database; the orientations of the images were changed (0°, 90°, 180° or 270°), as was the scaling technique, where the images were reduced to 75%, 50% and 25% of the size of the original images (Fig. 2). The images were resized to 200 × 200 pixels to allow analysis by the algorithm. All of these images were saved in a single database.

### Kernel application before the template

**Figure 4:**
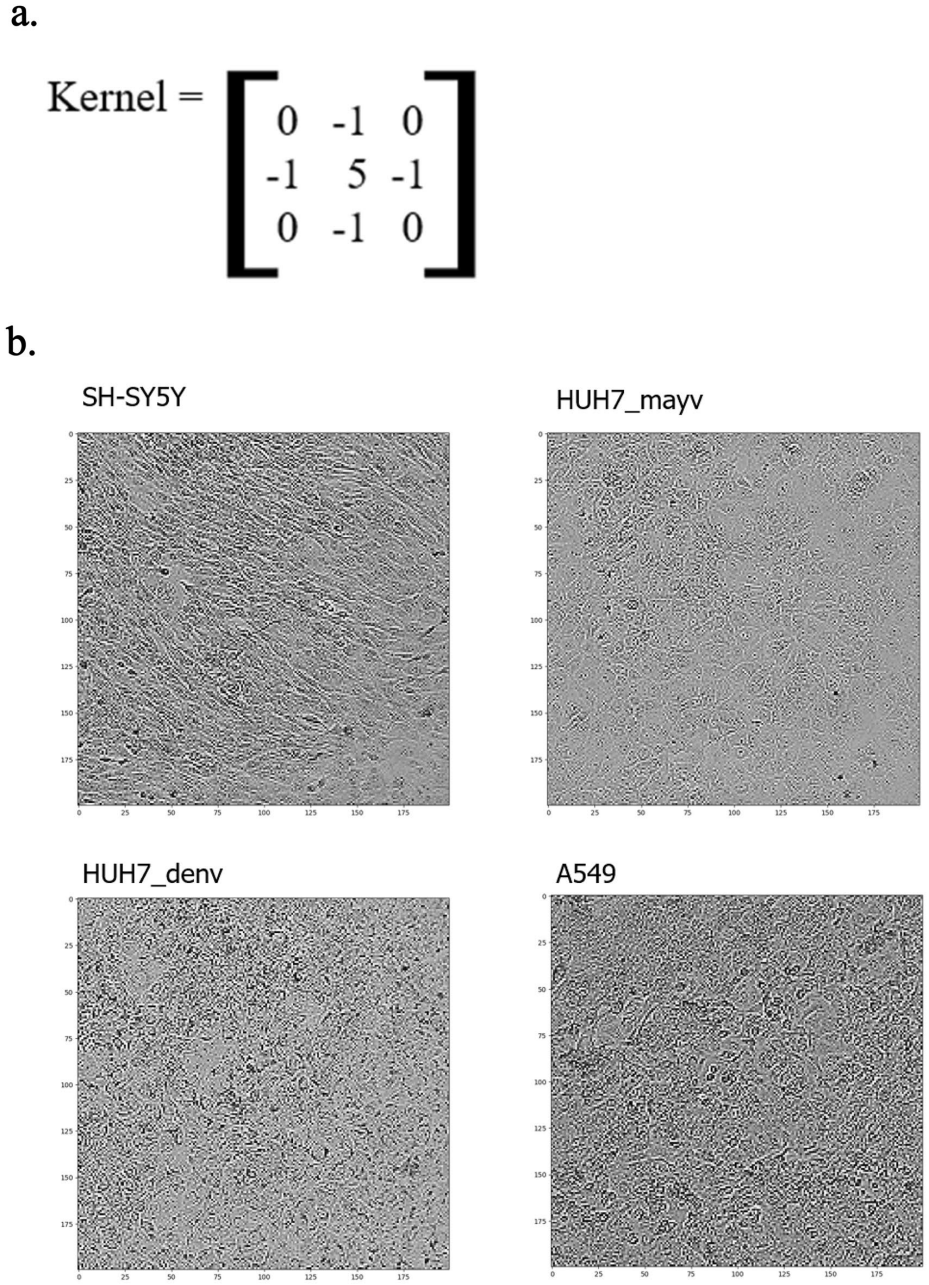
Adding Kernel to image preprocessing. (**a**) Kernel Sharpen applied to images. (**b**) Images of SH-SY5Y, HUH7_mayv, HUH7_denv and A549 strains after kernel application.

There was some homogeneity among the images, and the model sometimes found it difficult to differentiate between them. To work around this situation, filters were applied to highlight some of the most relevant characteristics of several images. This was only performed for the SH-SYS5, HUH7_mayv, HUH7_denv, and A549 lineages (Fig. 4). Several kernels were tested, and it was found that the best results were obtained with the Sharpen kennel, which accentuated the edges of the image. It adds contrast to edges, accentuating light and dark areas from a 3 × 3 matrix, similar to the edge detection kernel with a core value of 5^28^^.^

### Model validation

For CNN validation, 10% of the images were randomly removed, and the remaining 90% were used for training and testing. Of these images, approximately 70% were used to train the CNN, and 30% were used to test it. Table 2 shows the number of images of each bank.

**Table 2:** Separate number of images for each bank.

Cell line	Number of images for validation (10%)	Number of images for training/testing (90%)	Total image bank	Number of images for training (70%)	Number of images for testing (30%)
A549	113	1.023	1.136	716	307
HUH7_denv	152	1.368	1.520	957	411
3T3	84	748	832	523	225
VERO6	179	1.613	1.792	1.129	484
THP1	169	1.527	1.696	1.068	459
SH-SY5Y	116	1.051	1.168	736	316
A172	168	1.515	1.683	1.060	455
HUH7_mayv	128	1.152	1.280	806	346
Sum of data	1.109	9.997	11.107	6.995	3.003

### Classification model

The images were saved and identified with the name of their lineage. To create the classes, the name of each lineage was replaced with an integer value and used to create categorical classes ranging from 0 to 7.

#### Model evaluation based on accuracy metrics

Four possible outcomes were considered to evaluate the accuracy of the classification model. These were the true positives (TP), false positives (FP), true negatives (TN), and false negatives (FN).

##### Confusion matrix

The Confusion Matrix measures the number of correct classifications of the model in relation to the total of observations. *TPi corresponds to the number of false positives in class i.*

*N* is the total number of observations.

$$\frac{(TP1+TP2+\dots +TPn)}{N}$$

##### Precision

The precision is the number of correct classifications of the model in relation to the total of observations.

#### FNi corresponds to the number of false negatives in class i.

$$\frac{TPi}{(TPi+FNi)}$$

##### Recall

The recall is the ratio of true positives to the total positive observations in the class.

FNi corresponds to the number of false negatives in class i.

$$\frac{TPi}{(TPi+FNi)}$$

##### F1-Score

The F1-score is the harmonic mean of precision and recall, which seeks to balance the two metrics in unbalanced models.

$$\frac{2*Precision*Recall}{(Precision+Recall)}$$

##### ROC curve

The ROC (Receiver Operating Characteristic Curve) is the graphical representation of the performance of the classification model in relation to its true positives (True Positive Rate (TPR) and false positives (FPR). The ROC curve is then constructed by plotting the TPR as a function of the FPR at different classification threshold values.

$$TRP= \frac{TPi}{(TPi+FNi)}$$

$$FRP=\frac{FPi}{(FPi+TNi)}$$

### Regression model

As a target, the number of cells corresponding to each image from the HCS was recorded. This was used as the observed value, which was reduced in the same proportion of the images to perform the supervised training of the models and, subsequently, to perform the tests against the predicted values.

#### Model evaluation based on accuracy metrics

The Mean Absolute Error (MAE), Mean Square Error (MSE), and R2Score were used to evaluate the capacity and degrees of correct answers and errors of the models. However, during the training of the model, only MSE was used.

MSE is the $\frac{1}{n} \Sigma_{i=1}^{n}$ the squares of $\left( {Y_{i} - \hat{Y}_{i} } \right)^{2}$

$$MSE=\frac{1}{n}\Sigma {\left(y-\widehat{y}\right)}^{2}$$

### CNN

The first layer (Conv2D) was fitted with kernel_size = 3, and the activation function Rectified Linear Unit (ReLU), although other activation functions (LeakyReLU, Tahn, and Sigmoid) were tested, ReLU had the best performance. The same parameters were used in the sequence in the MaxPooling2D layers, ending with softmax output of eight classes. The same settings were used for the regression models, and the network's last layer was changed, ending with only one output neuron, with the ReLU activation function, which represents the number of cells in the image. To summarize the model information, the model.summary() method was used (Table 3).

**Table 3:** Parameters for CNN architecture development.

Layers	4
Kernel size	3
Activation funcion	ReLU
Pool size	2,2
Strides	2,2
Padding	Valid
Dropout	90%

## Data Availability

Ferreira, Eloiza KGD. and Silveira, GF. 2023. “Data-Analysis-Laboratory/Microscopy-Image-Analysis-Classification-Script-Article: 1.0.0”. Zenodo. 10.5281/zenodo.8415315, accessible at the link: https://zenodo.org/badge/latestdoi/701446984.
